# Zhongyong Thinking Style and Resilience Capacity in Chinese Undergraduates: The Chain Mediating Role of Cognitive Reappraisal and Positive Affect

**DOI:** 10.3389/fpsyg.2022.814039

**Published:** 2022-06-20

**Authors:** Shisi Zhou, Xueping Li

**Affiliations:** ^1^Department of Psychology, Institute of Education, China West Normal University, Nanchong, China; ^2^College of Preschool and Primary Education, China West Normal University, Nanchong, China

**Keywords:** Zhongyong thinking, resilience, culture, cognitive reappraisal, positive effect

## Abstract

Previous studies have suggested that the Zhongyong thinking style (influenced by Chinese culture) is associated with psychological features. However, little is known about the direct association between Zhongyong thinking and resilience and the underlying mechanisms of this relationship in Chinese culture. The present study aimed to investigate the association between Zhongyong thinking and undergraduates’ resilience and to assess whether cognitive reappraisal and positive effects mediated this association. A sample of undergraduates (*n* = 1,356, 70.4% female, mean age = 19 years) was recruited for this study and the participants completed the Zhongyong Thinking Style Scale (ZYTS), the Emotion Regulation Questionnaire (ERQ), the Positive Affect and Negative Affect Scale (PANAS), and the Resilience-11. Results indicated that the Zhongyong thinking style was positively and significantly associated with resilience. Undergraduates’ resilience was affected by Zhongyong thinking partly through 3 different pathways: the mediating role of cognitive reappraisal, the mediating role of positive effect, and the mediating chain role of both cognitive reappraisal and positive effect. These findings might provide a deeper understanding of the protective factors for resilience among Chinese undergraduates.

## Introduction

Resilience has been a focus of research in psychological and behavioral sciences in the last decade. Most previous conceptual approaches to understand resilience have considered it to be an individual trait, regarding resilience as a predisposition to succeed (Ungar, 2013). However, those researches fail to acknowledge the various influential factors, such as historical, social, and cultural influences on indigenous communities (Kirmayer et al., 2011). These factors connected with resilience are constructed from original cultural knowledge, indigenous philosophies, and beliefs (Thomas et al., 2015). Despite the various definitions of resilience, resilience can be recognized as an essential aspect of a better psychological and physical state (Smith et al., 2008; Osório et al., 2016), which can help individuals maintain mental health and fight depressive symptoms, anxiety, and other emotions (Iimura, 2022; Lau, 2022). Chinese undergraduates have been concerned a lot about the high incidence of mental health problems (Jiang et al., 2015). It is necessary to explore the relationship between resilience and the influential factors among Chinese undergraduates.

Zhongyong thinking is the most influential thinking style in China that originated from Chinese traditional philosophical culture, like Confucianism, and it initially functioned as a supreme morality and then evolved into a basic cognitive principle that Chinese people use to confront society (Chiu, 2000; Yang et al., 2016). For instance, Wu and Lin (2005) defined Zhongyong thinking as considering things from multiple aspects and making appropriate decisions for the whole situation. Studies have implied that Zhongyong thinking can influence people’s resilience under Chinese background (Cheng, 2009; Zheng et al., 2020). So Zhongyong thinking as a cultural-related variable that may connect with resilience needs to be investigated.

In addition, a few studies have reported some factors that predict the level of resilience, and the factors were also related to Zhongyong thinking. Guo and Zeng (2012) proved that individuals with high Zhongyong thinking tend to show greater cognition reappraisal. Yang et al. (2016) demonstrated that Zhongyong thinking played an important role in maintaining subjective well-being among contemporary Chinese young adults. Moreover, research revealed that individuals with high cognition reappraisal tend to show high resilience (Holl et al., 2017), and also positive effect is found correlated with resilience (Dewi and Ruidahasi, 2020).

No doubt, these previous studies have greatly enriched our understanding of resilience and its antecedents are observed. Individuals who maintain high Zhongyong thinking under the Chinese culture background are often likely to use the cognition reappraisal strategy and keep inner harmonious to be a positive emotional state, while cognition reappraisal and positive effect are essential factors for cultivating resilience. However, few studies verified the direct relation between Zhongyong thinking and resilience, and we know less about the mediators in the association. To bridge this gap, we explore the effect of Zhongyong thinking on resilience, and we sought to assess the role of cognition reappraisal and the positive effect between Zhongyong thinking and resilience in a sample of mainland Chinese undergraduates.

### Zhongyong Thinking and Resilience

Deeply influenced by the Chinese traditional philosophical traditions, including Confucianism, Chinese culture has had a distinctive morality and value system from the earliest times to the present day. With the development of cultural psychology, Spencer-Rodgers et al. (2010) explained that the thinking styles have cultural differences, especially between the West and the East. Many researchers have attempted to define the thinking style of the East, and they focused on the reconciliation of the two perspectives and the acceptance of contradictions (Peng and Nisbett, 1999). Holistic thinking and dialectical thinking were listed in these researches which emphasize on comparing East Asians and Westerners (Nisbett and Miyamoto, 2005; Choi et al., 2007; Spencer-Rodgers et al., 2010; Zhang et al., 2015). These thinking styles, to some extent, may relate to Zhongyong thinking, but the foundational theory and the starting point of the constructs were different (Please see the Note in the end for further discussion). China is a cultural and historical country in East Asia, and the effect of traditional Chinese culture and religion was not immutable and stationary. Zhongyong was known as a kind of high standard morality in ancient times. With the development of psychology, researchers have found that Zhongyong thinking is a system that involves values, behaviors, and perceptions, and people decide how to choose, execute, and correct their actions depending on this system (Yang et al., 2016). The concept of Zhongyong thinking is widely used in China. When Wu and Lin (2005) studied about Zhongyong thinking, they defined Zhongyong thinking as a process that takes situations into account from multiple aspects and accountable decisions are made for both personal feelings and the feelings of others considering different views. Therefore, the three features (multiple thinking, holism, and harmoniousness) are included. It is well established that Zhongyong thinking is related to individuals’ mental health under Chinese culture background, and Yang et al. (2016) demonstrated that Zhongyong thinking was significantly associated with an emotion system in a sample of 8,278 Chinese students.

The study of resilience has gone through a long process accompanied by many different views. Some perspectives define resilience as a trait that is comparatively stable and present in an individual at birth (Connor and Davidson, 2003; Lucken and Gress, 2010). The concept that resilience is like a skill or a quality people can develop and cultivate has also drawn much attention in the literature (Buzzanell, 2010). Others emphasize the social ecological understanding of resilience which is nested in various spheres of culture, political processes, family structure, and the community (Leadbeater et al., 2005; Ungar, 2013). Based on the social ecological theory of resilience, the factors that are congruent with cultural norms are important. Resilience is an important factor in advancing individuals’ mental health. For instance, research suggests that resilience may help individuals to deal with the negative psychological effects of traumatic events, including the Covid pandemic (Liao et al., 2021). It is therefore necessary to advance theory development about resilience and the relation between resilience and influential factors.

The correlation between Zhongyong thinking rooted in Chinese traditional culture and resilience should not be ignored. From Cheng’s (2009) research, we know that the thinking style influenced by Chinese traditional culture in China has a positive relationship with coping flexibility, and individuals need to have flexibles cognition appraisals in coping with different stressful events. Furthermore, it has been reported that the thinking style rooted in Chinese traditional culture significantly mediates the relationship between culture and resilience (Zheng et al., 2020). The belief that Zhongyong thinking may effect resilience capacity is implied in these findings.

According to the studies, it can be predicted that Zhongyong thinking should play a crucially effective role in promoting resilience in China.

### Cognitive Reappraisal as a Mediator

Emotion regulation refers to the process when the emotion arouses, maintains, and recovers individual uses to influence the occurring, experiencing, and expressing of emotion (Gross, 2000; Gross, 2007). Cognitive reappraisal is a strategy that individuals often selectively reinterpret events by changing the subjective appraisals to reframe an emotional stimulus (Gross and John, 2003). Previous experimental evidence shows that social cognition has a substantial impact on an individual’s emotion regulation (Westerhof-Evers et al., 2019), and the differences in emotion regulation strategies exist in the different cultures (Miyamoto et al., 2014; Ip et al., 2021). Meanwhile, the thinking style rooted in eastern culture as a basic cognition has drawn lots of attention (Spencer-Rodgers et al., 2010). Evidence showed that Zhongyong thinking significantly correlates with cognitive reappraisal strategy under the Chinese cultural background (Guo and Zeng, 2012). Literature suggests that cognitive reappraisal correlated with healthier emotion and better well-being (Cutuli, 2014).

*Cognitive reappraisal* is an effective emotion regulation strategy which is an essential aspect to enhance resilience. Many findings have supported that an individual’s emotion regulation strategy was correlated with resilience (Mestre et al., 2017; Vaughan et al., 2019). For instance, Zhang et al. (2020) evaluated the relationship between resilience and emotion regulation among preschool left-behind children. The results revealed that children with higher cognitive reconstruction had a lower risk of low resilience. Moreover, studies showed that the cognitive reappraisal strategy could serve as a path to explain resilient development among mentally healthy individuals with and without experience of childhood abuse and neglect (Holl et al., 2017).

In summary, we assume that the more the individuals with a higher Zhongyong thinking style obtain under cultural background, the more cognitive reappraisal strategy the individuals may use. Furthermore, more use of cognitive reappraisal promotes higher resilience, suggesting that cognitive reappraisal may function as a mediator in the association between Zhongyong thinking and resilience.

### Positive Effect as a Mediator

Another potential mediator in the association between Zhongyong thinking and resilience is positive effect which is an essential feature of subjective well-being and mental health. Positive effect is defined as individuals’ propensity to experience positive emotions and deal with challenges and interpersonal relationship in a positive way (Lopez et al., 2018). Lots of studies showed that positive effect could predict or promote a large number of desirable outcomes besides resilience (Davidson et al., 2010; Rackoff and Newman, 2020). For example, Buchanan (2014), who first pointed out that there were protective factors to promote the development of resilience and risk ones, stated that positive emotions like well-being, inner calm, especially experienced in early childhood, could help children achieve resilience. Furthermore, as revealed by Dewi and Ruidahasi (2020), maintaining positive effect could enhance resilience in the rehabilitation institution.

When we focus on Zhongyong thinking and emotion, the “zhong” and “he” always draw our attention. “Zhong” refers to mater the extremes but deploy the mean, and “he” is related to the aspiration of the harmonious and coexistent directions (Wu and Lin, 2005). However, the opinion Zhongyong thinking encourages the characteristic of “finding the good in the bad” is less mentioned, and this characteristic can promote individuals’ positive emotion. In Yang et al.’s (2016) longitudinal studies, the training of Zhongyong thinking in group psychotherapy to reduce Chinese students’ depression symptoms was approved. Moreover, cross-sectional studies revealed that higher level of Zhongyong thinking was interrelated with fewer depressive and anxiety symptoms (Zhan et al., 2013).

Underpinning these works, we assume that the higher Zhongyong thinking level Chinese college students have, the more positive effect they may obtain and this results in a more positive effect which can lead to more resilience. Therefore, it is reasonable to infer that positive effect may also mediate the association between Zhongyong thinking style and Chinese college students’ resilience.

### Cognitive Reappraisal and Positive Effect

It is known that emotion regulation plays a crucial part in influencing an individual’s psychological and health problems (Ramirez-Ruiz et al., 2020). Deficits in regulating emotion strategies may lead to disorders in psychology and psychiatry (Pappaianni et al., 2020). From a systematic emotion-regulation strategies view, tons of published researches focused on maladaptive emotion regulation strategy, and evidence showed the strategy was positively associated with and anxiety, depression, and stress (Ramirez-Ruiz et al., 2020). While the association between emotion regulation and positive psychological concepts had been given less attention in the literature (Ramirez-Ruiz et al., 2020), even positive psychology came a long way. Fortunately, there were still some researches that could be listed. Schanowitz and Nicassio (2006) found that more use of positive reappraisal can predict higher positive effect. Exploring the relationship between perceived stress and positive effect, Teixeira et al. (2021) presented evidence that functional cognitive reappraisal had a mediating effect on the association.

Following these studies, we posit that cognitive reappraisal is directly related to positive effect, which subsequently improves resilience.

### The Current Study

It is the first study to directly investigate the relationship between Zhongyong thinking and resilience although researches have implied the culture-related factor connected with resilience. Also it has been suggested that although cognitive reappraisal and positive effect connect with Zhongyong thinking and resilience, the underlying mechanisms of the relationship between Zhongyong thinking and resilience remains unclear. We took efforts to understand deeper the relationship between Zhongyong thinking and resilience and sought to expand the literature by specifying the mechanisms underlying the association between Zhongyong thinking and resilience by considering the mediating effect of cognitive reappraisal and positive effect.

In summary, in this study we investigated the relationship between Zhongyong thinking and resilience and tested the mediating effects of cognitive reappraisal and positive effect in this relationship using a sample of Chinese undergraduates. Considering the previous empirical researches and theoretical studies, we proposed four hypotheses: (1) Zhongyong thinking significantly connect with resilience; (2) cognitive appraisal mediate the relationship between Zhongyong thinking and resilience; (3) positive effect mediate the relationship between Zhongyong thinking and resilience; (4) cognitive appraisal and positive effect play a chain mediating effect on the relationship between Zhongyong thinking and resilience.

## Materials and Methods

### Participants

The participants of this study were undergraduates who came from different provinces in China. A sample of 1,382 college students was recruited from three universities in mainland China. Excluding 16 uncompleted questionnaires (missing items were more than 15% of the total items) and 10 unreasonable answers, 1,356 (*n* = 1,356) valid questionnaires comprised the study sample (valid response rate was 100%). They ranged in age from 18 to 26. In this sample, 70.4% of the participants were women and 29.6% of the participants were men. The Han nationality was 1,294 (95.4%) and the minorities were 61 (4.5%).

### Procedure

The Institutional Review Boards (IRBS) approved the present research to begin the study. After research administrator orally explained the same instruction on how to manage the questionnaires and expounded the purpose of the present study, all students took part in this survey voluntarily in the classroom. To protect their personal information, we collected the data anonymously. The effectiveness of data collection was ensured. Each participant was paid 3RMB payments for their participation. Altogether, the instruments took approximately 30 min to complete.

### Measures

#### Zhongyong Thinking Style

The Zhongyong thinking Style Scale (ZYTS; Wu and Lin, 2005) was used to measure participants’ Zhongyong thinking levels. Three dimensions of Zhongyong thinking are measured on the 13-item scale. They are multi-thinking, holism, and harmoniousness. The items were hypothetical opinion–expression situations and participants needed to evaluate their thinking process in these situations. Here are some examples of the items: “When discuss with others I will thinking about the conflicting opinions from others” “I always consider things from multiple aspects” (Multi-thinking); “I will try to find a balance between others’ views and my own opinion” “I will adjust my thoughts after taking into account others’ suggestions “(Holism); “When making decisions, I will take the atmosphere of harmoniousness into account”(Harmoniousness). Each item is rated on a 7-point Likert scale (1 = strongly disagree, 7 = strongly agree). Item scores are summed to yield a total score ranging from 7 to 91, with higher scores demonstrating higher Zhongyong thinking. Participants were invited to evaluate their thinking process in a The ZYTS has been widely used among the Chinese and has shown good reliability and validity (Hu et al., 2012; Sun et al., 2014), and the internal consistency (Cronbach’s alpha) of this study was 0.80.

#### Resilience

Participants completed the Chinese version of Resilience-11 (Gao et al., 2013) to assess an individual’s resilience. The RS-11 is translated and modified from the original English version of RS-11 (Wagnild and Young, 1993). It is an 11-item tool, and each item is rated on a 7-point Likert scale. Total scores range from 11 to 77, and higher scores demonstrate higher levels of resilience. Good reliability and validity of the revised Chinese version have been tested and shown for Chinese samples, and Cronbach’s alpha coefficient is 0.83.

#### Emotion Regulation Questionnaire

In this study, the emotion regulation was measured by the Emotion Regulation Questionnaire (Gross and John, 2003), which consists of two dimensions, cognitive reappraisal, and expression suppression. The Chinese version of the revised emotion regulation questionnaire has been previously validated (Wang et al., 2007). This scale is a 10-items, 7-point Likert-type self-report instrument aimed to evaluate the participants’ inclinations to regulate their emotions. The higher the score is the more the frequency of emotion regulation strategy the people use. The reliability coefficients of the dimensions of cognitive reappraisal and expressive suppression are 0.85 and 0.77 (Wang et al., 2007). In the current study, the Cronbach’s alpha coefficients were 0.92 for cognitive reappraisal, 0.84 for expression suppression, and 0.92 for the whole emotion regulation questionnaire.

#### Positive Effect and Negative Effect Scale

The positive effect and negative effect scale (PANAS) is a self-report questionnaire with 20 emotion items that have been used to measure positive effect (PA) and negative effect (NA). Participants had to indicate the extent to which they have felt each effect (e.g., “active” and “hostile”) using a 5-point Likert scale. The Chinese version of the scale has shown high internal consistency, and adequate internal consistency and validity have been demonstrated in lots of previous studies (Huang and Yang, 2003; Zhang et al., 2004). In this study, the Cronbach’s alpha coefficients for the positive effect and negative effect sub-scale were 0.91 and 0.81, respectively.

### Analytical Methods

We conducted statistical analyses by using SPSS (version 21.0) and AMOS (version 24.0). First, Pearson correlation was tested to investigate the association between Zhongyong thinking, cognitive reappraisal, positive effect, and resilience. Multiple comparisons were corrected using a FDR method with a corrected threshold of *q* < 0.05. Second, we conducted serial mediation analysis with the bootstrapping method, in which the indirect effect of Zhongyong thinking on resilience through cognitive reappraisal, through positive emotion, and through both cognitive reappraisal and positive emotion was tested. This bootstrapping analysis with 5,000 iterations was conducted using PROCESS Macro (Preacher and Hayes, 2008) to test the significance of the indirect effect of the mediator. It was believed that the absence of zero in the confidence interval (CI) indicates the significance of the point estimate (*p* < 0.05; Hooper et al., 2008). Third, a series of hierarchical multiple regressions were conducted in Zhongyong thinking and resilience. The standardized predictive variable and responding variable (Zhongyong thinking and resilience) were required in a regression equation. The incremental change in *R*^2^ and *F-*value was used to evaluate the main effect of the study variables.

## Results

### Preliminary Data Analyses

Univariate and multivariate normality was assessed by the values of skewness and kurtosis. Skewness values ranged from −1.14 to 0.01 and kurtosis values ranged from 0.34 to 2.73 (for Zhongyong thinking, cognitive reappraisal, positive effect, and resilience, respectively), which indicated that there was no severe violation of normal distribution (Sk < | 3| and Ku < | 10|; Kline, 2005).

### Common Methods Bias Analyses

Common method deviation might occur since all the collected questionnaires were from university students’ self-reports. The Harman single factor method was conducted in this study so that the common methodological deviations can be tested and avoided. The results showed that there were 8 factors whose characteristic value was greater than 1, and the interpretation rate of the first factor was 24.86%, less than 40%. Hence, the influence of common method deviation in the questionnaires collected in this study can be excluded.

### Bivariate Correlations Between Variables of Interest

As shown in Table 1, significant correlations were found between Zhongyong thinking, cognitive reappraisal, positive effect, and resilience. After FDR adjustment, Zhongyong thinking were significantly and positively correlated with resilience (*r* = 0.49, *p* < 0.01, q of FDR < 0.05), cognitive reappraisal (*r* = 0.46, *p* < 0.01, q of FDR < 0.05), and positive effect (*r* = 0.28, *p* < 0.01, q of FDR < 0.05). Cognitive reappraisal was positively related to positive effect (*r* = 0.38, *p* < 0.01, q of FDR < 0.05) and resilience (*r* = 0.61, *p* < 0.01, q of FDR < 0.05). Moreover, positive effect was positively correlated with resilience (*r* = 0.53, *p* < 0.01, q of FDR < 0.05).

**TABLE 1:** Descriptive statistics and bivariate correlation (*n* = 1,356).

	*Mean*	*SD*	1	2	3
1. Zhongyong thinking	68.3	11.6	**−**		
2. Cognitive reappraisal	29.0	5.6	0.46**	**−**	
3. Positive effect	30.8	5.8	0.28**	0.38**	**−**
4. Resilience	52.7	8.3	0.49**	0.61**	0.53**

*SD, standard deviation.*

**p < 0.01.

### The Chain Mediation Effects Analyses

There were three equations which were used to test the mediating role of the cognitive reappraisal and positive effect in the relationship between Zhongyong thinking and resilience. As shown in Table 2 Zhongyong thinking had a directly and positively significant impact on the level of undergraduates’ resilience (β = 0.16, *p* < 0.001) in equation 3, cognitive reappraisal (β = 0.23, *p* < 0.001) in equation 1, and positive effect (β = 0.07, *p* < 0.001) in equation 2. Furthermore, there was a significant direct prediction from cognitive reappraisal to positive effect (β = 0.33, *p* < 0.001) in equation 2. Finally, cognitive reappraisal (β = 0.55, *p* < 0.001) and positive effects (β = 0.47, *p* < 0.001) could predict the resilience positively and significantly in equation 3. Based on the theory of Rosnow and Rosenthal (1996), the results of Cohen’s Standard, *d* and *R*^2^ in Table 3 showed that equation 3 had a large effect.

**TABLE 2:** Multiple linear regression results for testing the mediating role of cognition reappraisal and positive effect in the relationship between Zhongyong thinking and resilience (*n* = 1,356).

Predictor variable	Outcome variable	*R*	*R* ^2^	*F*	β	*t*	Boot LLCI	Boot ULCI
**Equation 1**								
Zhongyong thinking	Cognitive reappraisal	0.47	0.22	373.00	0.23	19.31***	0.205	0.248
**Equation 2**								
Zhongyong thinking	Positive effect	0.40	0.16	127.79	0.07	4.74***	0.040	0.094
Cognitive reappraisal					0.33	11.32***	0.271	0.385
**Equation 3**								
Zhongyong thinking	Resilience	0.72	0.51	476.67	0.16	10.30***	0.129	0.189
Cognitive reappraisal					0.55	16.80***	0.490	0.619
Positive effect					0.47	15.98***	0.414	0.530

*The 95% confidence intervals do not overlap with zero.*

*BootLLCI and BootULCL were 95% confidence interval lower and 95% confidence interval upper calculated by the bias-corrected bootstrap method for testing indirect effects.*

***p < 0.001.

**TABLE 3:** Indirect effect of cognitive reappraisal and positive effect (*n* = 1,356).

	Effect	Boot SE	Boot LLCI	Boot ULCI	Ratio of indirect to total effect	Ratio of indirect to direct effect
Total indirect effect	0.19	0.014	0.164	0.220	54%	120%
Indirect effect 1	0.13	0.011	0.102	0.147	36%	78%
Indirect effect 2	0.03	0.004	0.026	0.045	9%	22%
Indirect effect 3	0.03	0.007	0.018	0.046	9%	20%

*Indirect effect 1 was Zhongyong thinking→cognitive reappraisal→resilience.*

*Indirect 2 was Zhongyong thinking→positive effect→resilience.*

*Indirect 3 was Zhongyong thinking→cognitive reappraisal→positive effect→resilience.*

*Boot SE, Boot LLCI, and Boot ULCL were estimated standard error, 95% confidence interval lower and 95% confidence interval upper through bias-corrected percentile bootstrap method used for testing indirect effects.*

*The 95% confidence intervals did not overlap with zero.*

**FIGURE 1:**
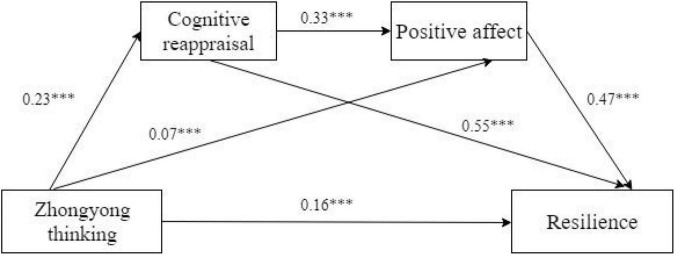
The chain mediating effect of cognitive reappraisal an positive affect. ***p < 0.001.

Table 3 and Figure 1 show the results of the chain mediating effect of cognitive appraisal and positive effect. The total indirect effect was 0.19 and accounted for 54% of the total effect (0.35) and 120% of the direct effect (0.15) in the relationship between Zhongyong thinking and resilience. The indirect mediating effects of cognitive appraisal and positive effect on the relationship between Zhongyong thinking and resilience were significant and there were three different pathways contained in the total indirect effects. According to the indirect effects 1,2 and 3 in Table 3, we found that Zhongyong thinking influenced the resilience of Chinese undergraduates partly through the mediator of cognitive reappraisal, through the mediating function of positive effect and through the chain mediating role of both cognitive reappraisal and positive effect. Moreover, it was, respectively, accounted for 36%, 9%, and 9% of total effect by indirect effects 1,2, and 3. The 95% CI did not include zero, confirming all significant indirect effects.

Furthermore, three equations testing and comparing the mediating effects of cognitive appraisal and positive effect in the relationship between Zhongyong thinking and resilience were used in this study. Table 4 shows that Zhongyong thinking could directly and significantly positively predict resilience (β = 0.16, *p* < 0.001) of Chinese undergraduates in equation 3, cognitive appraisal (β = 0.22, *p* < 0.001) in equation 1, and positive effect (β = 0.14, *p* < 0.001) in equation 2. In addition, cognitive appraisal (β = 0.55, *p* < 0.001) and positive effect (β = 0.47, *p* < 0.001) had a significant and positive predictive power on resilience in equation 3. Based on the theory of Rosnow and Rosenthal (1996), the results of Cohen’s standard, *d* and *R*, in Table 3 showed that equation 3 had a large effect.

**TABLE 4:** Multiple linear regression results for testing the mediating role of cognitive reappraisal and positive effect in the relationship between Zhongyong thinking and resilience (*n* = 1,356).

Predictor variable	Outcome variable	*R*	*R* ^2^	*F*	β	*T*	Boot LLCI	Boot ULCI
**Equation 1**								
Zhongyong thinking	Cognitive reappraisal	0.46	0.22	373.00	0.22	19.31***	0.20	0.25
**Equation 2**								
Zhongyong thinking	Positive effect	0.28	0.07	116.60	0.14	10.80***	0.12	0.17
**Equation 3**								
Zhongyong thinking	Resilience	0.72	0.51	476.67	0.16	10.30***	0.13	0.19
Cognitive reappraisal					0.55	16.80***	0.49	0.62
Positive effect					0.47	15.98***	0.41	0.53

*95% confidence intervals do not overlap with zero.*

*Boot LLCI and Boot ULCL were 95% confidence interval lower and 95% confidence interval upper calculated by the bias-corrected bootstrap method for testing indirect effects.*

*The alternative chain model of Zhongyong thinking→positive effect→cognitive reappraisal→resilience was also significant, but considering the result of the parallel results, the effect-regulation model, and the length of the article, this study only concentrated on the chain model of Zhongyong thinking→cognitive reappraisal→positive effect→resilience rather than the alternative chain model.*

**FIGURE 2:**
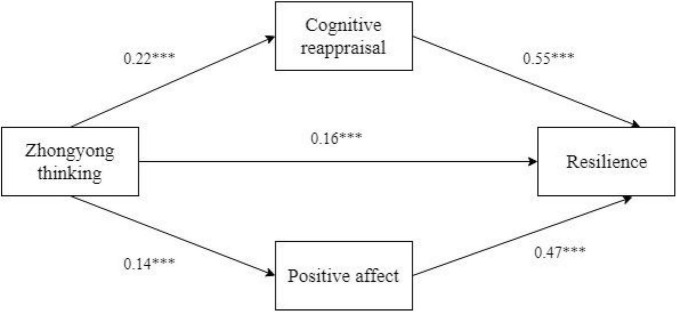
The mediating effect of cognitive reappraisal and positive effect in a parallel model. ***p < 0.001.

Table 5 and Figure 2 show the results of comparing the mediating effect of the cognitive reappraisal and positive effect in a parallel model. The indirect effect of cognitive appraisal was 0.06, accounting for 35% of the total effect (0.35) and 78% of the direct effect (0.16) in the association between Zhongyong thinking and resilience. The indirect effect of the positive effect was 0.13 and it accounted for 19% of the total effect (0.35) and 42% of the direct effect (0.16). The 95% CI did not include zero, confirming all significant indirect effects. Therefore, the indirect effect of positive effect (0.13) was stronger than that of cognitive reappraisal (0.06), which meant both cognitive reappraisal and positive effect were considered to be mediators of Zhongyong thinking in resilience, and the positive effect played a more important role than cognitive reappraisal.

**TABLE 5:** The comparison of the mediating effect of the cognitive reappraisal and positive effect in the relationship between Zhongyong thinking and resilience (*n* = 1,356).

	Effect	Boot SE	Boot LLCI	Boot ULCI	Ratio of indirect to total effect	Ratio of indirect to direct effect
Total effect	0.35	0.02	0.308	0.392	−	−
Direct effect	0.16	0.02	0.123	0.194	−	−
Total indirect effect	0.19	0.01	0.165	0.220	54%	120%
Mediating effect of CR	0.06	0.01	0.104	0.148	35%	78%
Mediating effect of PA	0.13	0.01	0.052	0.082	19%	42%

*CR, cognitive appraisal; PA, positive effect.*

*Mediating effect of CR was Zhongyong thinking→cognitive reappraisal→resilience.*

*Mediating effect of PA was Zhongyong thinking → positive effect → resilience.*

*Boot SE, Boot LLCI and Boot ULCL were estimated standard error, 95% confidence interval lower and 95% confidence interval upper through bias-corrected percentile bootstrap method used for testing indirect effects.*

*The 95% confidence intervals did not overlap with zero.*

## Conclusion

There were two purposes of the present study. Firstly, to investigate whether Zhongyong thinking was a significant predictor of resilience among Chinese undergraduates. Secondly, to explore the crucial role of cognitive reappraisal and the positive effect on the relationship between Zhongyong thinking and resilience in a sample of Chinese undergraduates. The results of multiple linear regressions in this study showed that Zhongyong thinking is positively related to resilience. Mediation analysis indicated that not only cognitive appraisal but also positive effect could partly mediate the relationship between Zhongyong thinking and resilience, but also there is a chain mediating effect of “Zhongyong thinking–cognitive appraisal–positive effect–resilience.”

The preliminary evidence showed that Zhongyong thinking had a significant positive effect on resilience (as shown in Table 1), which was also approved in the mediation analysis in Table 5. This positive correlation between Zhongyong thinking and resilience consolidates the relationship between the two factors. Cheng (2009) reported that as individuals had a higher capacity for dialectical thinking, they tended to display more flexibility in coping with different stressful events. Research from a cross-cultural report has implied that the thinking style which was rooted in Chinese traditional culture may effect resilience capacity in the cultural context (Zheng et al., 2020).

Consistent with our expectations, the results showed that Zhongyong thinking influenced resilience *via* three pathways: cognitive reappraisal, positive effect, and the chain mediating effect of cognitive appraisal and positive effect, which benefits us to gain a deeper comprehension of the mechanism between Zhongyong thinking and resilience. First, the partial mediation role of cognitive reappraisal on the association between Zhongyong thinking and resilience is supported. The result of correlation analysis indicated that cognitive reappraisal had a significant, positive relationship with Zhongyong thinking and resilience. This result was consistent with previous studies that mentioned the thinking style, characterized as multi-thinking, holism, and harmoniousness, played an important role in influencing the usage of emotion regulation strategy (Yang and Li, 2014), the experience, and expression of emotion (Spencer-Rodgers, 2004). The prerequisite for mediation analysis related to the results of the correlation analysis was satisfied (Baron and Kenny, 1986). From the further investigation of the mediation role of cognitive appraisal on the relationship between Zhongyong thinking and resilience, the findings indicated that when students developed a high level of the culturally rooted Zhongyong thinking style, they were more likely to use the cognitive appraisal strategy which was positively associated with resilience. The possible reasons for it might be as follows: Zhongyong thinking not only emphasizes the interpersonal harmoniousness in daily life but also stresses the multithinking which is a tendency of considering various possibilities from multiple perspectives when making decisions or expressing opinions. This tendency may promote people to develop a cognition that is not stubborn or unmodifiable, and the cognition tendency is propitious to people using cognitive reappraisal strategy, which also needs changeable and not obstinate subjective appraisals to the emotionally concerning situation.

Our results not only supported the mediating role of cognition appraisal but also verified the mediating effect of positive effect underlying the Zhongyong thinking–resilience relationship. The complexity and contradiction of Chinese emotional experience influenced by Chinese culture and rooted in Chinese thinking style had been mentioned in many theoretical discussions (Goetz et al., 2008; Spencer-Rodgers et al., 2010), and there were a few statistical and experimental researches investigating the correlation between effect and Zhongyong thinking. Recently, a study focused on the emotional distress of Chinese college students provided that Zhongyong thinking correlated negatively with depression and anxiety (Hou et al., 2020), which partially showed the support for our finding. In addition, positive effect is a protector of resilience and mediates the relationship. Together, there was some evidence that positive effect can have a mediating role between Zhongyong thinking and resilience. These findings implied that Zhongyong thinking influenced resilience through 2 pathways: the effect of Zhongyong thinking on resilience mediated by cognition reappraisal and the effect of Zhongyong thinking on resilience mediated by positive effect.

Moreover, we also found another significant path of Zhongyong thinking→cognition reappraisal→positive effect→resilience. This mediation model illustrated that cognition reappraisal acted as a mediator between Zhongyong thinking and positive effect, while positive effect mediated the link between cognition reappraisal and resilience. There were statistical and experimental studies, which confirmed the findings that individuals who developed high Zhongyong thinking were likely to use the cognitive reappraisal strategy more frequently (Guo and Zeng, 2012), in return, more usage of cognitive reappraisal were associated with a higher level of positive effect (Wante et al., 2017). In addition, that positive effect plays a mediation role in the relationship between cognition reappraisal and resilience also can be supported. Based on the emotion regulation theory of Gross and John (2003), individuals with more usages of cognition reappraisal tended to experience more positive effect. The studies also supported the positive correlation between cognition reappraisal and positive effect (Yang and Li, 2014; Oikawa et al., 2017), while positive effect is a protective factor that can promote individuals’ resilience (Lord et al., 2015). Evidence of experiencing positive effect can mediate the relationship between adolescents’ perceived parenting styles, and resilience can also partially verify our finding (Nikmanesh et al., 2020). That is to say, the chain mediation effect of cognitive appraisal and positive effect indicated that Chinese undergraduates with a higher level of Zhongyong thinking would report more usage of cognition reappraisal, which may result in a higher level of positive effect and ultimately lead to an increased possibility of resilience.

Above all, the present study not only found that Zhongyong thinking could account for resilience but also explored the underlying mechanisms between Zhongyong thinking and resilience among Chinese undergraduates. These findings indicated that Zhongyong thinking affected undergraduates’ resilience partly through three different pathways: the mediator of cognitive reappraisal, the mediator of positive effect, and the chain mediating role of both cognitive reappraisal and positive effect. To our knowledge, this is the first time to investigate the mechanism in the relationship between Zhongyong thinking and resilience. Furthermore, the findings are useful for clinicians or psychotherapists working with Chinese undergraduates. Resilience is an important factor that promotes individuals’ mental health, considering the culture-related Zhongyong thinking in the therapeutic settings is also valuable for these undergraduates’ lack of resilience.

### Limitations and Future Direction

It is important to note the limitations of this study. First, the cross-sectional design was used in this study, so it may have an influence on revealing the casual associations among variables. In future studies, using a longitudinal design is helpful to supply a developmental perspective. The second limitation was that there may be other variables that acted as a mediator in the relationship between Zhongyong thinking and resilience. Even though there was a lack of research to imply that negative effect and suppression mediated the relationship, the fact that negative effect and suppression are related to resilience cannot be ignored. Factors that may function in the relationship should be further illustrated in future studies. Third, a cross-culture research is needed to investigate the relationship between Zhongyong thinking and resilience and mediating effects outside China to deeply understand the relationship from different aspects. Furthermore, the data obtained by self-report measurements may lack objectivity due to self-report bias and social desirability. Based on Chinese culture, Zhongyong thinking is a complex and dynamic thinking process, and there are opinions that it are doubtful to measure Zhongyong thinking by self-report questionnaires. However, Wu and Lin (2005) insisted that Zhongyong thinking is a conscious thinking process in which individuals could consciously balance the external information and internal demands and integrate a cultural-based behavioral criterion, and it is practicable to introspect and report by individuals. Future researchers could measure these variables in behavioral experiments or event recording methods.

Despite these limitations, the present study makes contributions that should not be ignored. This study is the first to explore the association between Zhongyong thinking and resilience among Chinese students. Compared with the personal trait theory, which argued individuals were born with resilience and shaped by different personal traits, the results of the study highlight the consideration of environmental and cultural factors to influence the development of resilience for Chinese college students. It is also worth mentioning our efforts, including exploring the mediating mechanisms or processes underlying the relationship between Zhongyong thinking and resilience, testing the paths from Zhongyong thinking, cognition reappraisal, and positive effect to resilience.

### Implications

As the number of domestic and international conflicts increased and the risk of disease and disaster grew, the incidence of psychological problems is likely to continue to rise. There are more opportunities for therapists and mental health professionals from diverse countries to exchange effective resources and communicate with each other because of the mixing and gathering of cultures. It is noteworthy that the cultivating of therapists and mental health professionals need to be aware of the function of traditional cultural heritage and well adapted to home culture so that they can provide appropriate care.

### Note

With the development of Culture Psychology, results of studies have proved that human mind is not universal cross-culture and the differences existing between Westerners and Easterners have drawn lots of attention in the past decades. Holistic thinking and dialectical thinking, which I mentioned in front, were listed in these culture-related researches because Zhongyong thinking also originates from culture, and some researches have mixed these concepts up. Holistic thinking and dialectical thinking, to some extent, may relate with Zhongyong thinking, but the foundational theory and start pointing of the constructs were different. First of all, for measuring Holistic thinking, Choi et al. (2007) developed Analysis–Holism Scale to compare East Asians and Westerners in a theoretical model of analytic vs. holistic thinking, and in Analysis–Holism Scale Choi et al. (2007) created a task such that one has to choose only one of the two alternative solutions to compare holistic and analytic thinking the two different thinking styles. But for Zhongyong thinking, a traditional Confucius interpersonal style with emphasis on interpersonal harmony and connect, the theoretical foundation of the scale benefits from Chinese traditional culture and philosophical thought without comparing different cultures, and in Zhongyong thinking Scale there are not alternative solutions in the items to compare different thinking styles, it only focuses on Zhongyong thinking itself. So these differences mean a lot to these concepts. Secondly, Spencer-Rodgers et al. (2010) call Easterners’ dialecticism naive dialecticism, which represents three aspects of Easterners’ minds. There are researchers using Dialectical Self Scale among Japanese to investigate culture differences (Zhang et al., 2015). As for Zhongyong Thinking Scale, it comes from Chinese traditional culture and there are no researches proved that it can be used in other countries for now. Thirdly, if Holistic thinking and dialectical thinking, to some extent, can express the thinking of East Asians, and China as a historical and cultural country in East Asia so that some characteristics of these concepts sounding like the same and some similarities existing in these concepts may be judged reasonable, differences of these concepts should not be ignored.

## Data Availability Statement

The original contributions presented in this study are included in the article/Supplementary Material, further inquiries can be directed to the corresponding author/s.

## Ethics Statement

The studies involving human participants were reviewed and approved by China West Normal University. All subjects gave written informed consent in accordance with the Declaration of Helsinki.

## Author Contributions

SZ: acquisition of data, investigation, analysis and interpretation of data, drafting the manuscript, and software. XL: critical manuscript revision, development or design of methodology, and creation of models. Both authors contributed to the article and approved the submitted version.

## Conflict of Interest

The authors declare that the research was conducted in the absence of any commercial or financial relationships that could be construed as a potential conflict of interest.

## Publisher’s Note

All claims expressed in this article are solely those of the authors and do not necessarily represent those of their affiliated organizations, or those of the publisher, the editors and the reviewers. Any product that may be evaluated in this article, or claim that may be made by its manufacturer, is not guaranteed or endorsed by the publisher.
